# What’s inside is all that counts? The contours of everyday thinking about self-control

**DOI:** 10.1007/s13164-021-00573-2

**Published:** 2021-09-30

**Authors:** Juan Pablo Bermúdez, Samuel Murray, Louis Chartrand, Sergio Barbosa

**Affiliations:** 1 Institut de Philosophie, Université de Neuchâtel, Espace Tilo-Frey 1, 2000 Neuchâtel, Switzerland; 2 Programa de Filosofía & Área de Investigación Salud, Conocimiento Médico y Sociedad, Universidad Externado de Colombia, Bogotá, Colombia; 3 Department of Psychology and Neuroscience, Duke University, Durham, NC USA; 4 Departamento de Psicología, Facultad de Ciencias Sociales, Universidad de los Andes, Bogotá, Colombia; 5 Center for Philosophy of Science, University of Pittsburgh, Pittsburgh, PA USA; 6 Departamento de Psicología, Universidad del Rosario, Bogotá, Colombia

**Keywords:** Self-control, Internalism, Willpower, Weakness of will, Folk psychology, Attention, Motivation

## Abstract

Does self-control require willpower? The question cuts to the heart of a debate about whether self-control is identical with some psychological process internal to the agents or not. Noticeably absent from these debates is systematic evidence about the folk-psychological category of self-control. Here, we present the results of two behavioral studies (*N* = 296) that indicate the structure of everyday use of the concept. In Study 1, participants rated the degree to which different strategies to respond to motivational conflict exemplify self-control. Participants distinguished between intra-psychic and externally-scaffolded strategies and judged that the former exemplified self-control more than the latter. In Study 2, participants provided various solutions to manage motivational conflict and rated their proposals on effectiveness. Participants produced substantially more intra-psychic strategies, rated them as more effective, and advised them at a higher rate than externally-scaffolded strategies. Taken together, these results suggest that while people recognize a plurality of strategies as genuine instances of self-control, purely internal exercises of self-control are considered more prototypical than their externally-scaffolded counterparts. This implies a hierarchical structure for the folk psychological category of self-control. The concept encompasses a variety of regulatory strategies and organizes these strategies along a hierarchical continuum, with purely intra-psychic strategies at the center and scaffolded strategies in the periphery.

**Supplementary Information:**

The online version contains supplementary material available at 10.1007/s13164-021-00573-2.

## Introduction

Self-control consists in the ability to align one’s behavior with one’s commitments in the face of contrary motivations. Philosophers and cognitive scientists disagree about the kind of alignment between behavior and commitment that constitutes exercises of self-control. Some claim that the alignment between behavior and commitment must result from a distinctive type of mental process, such as willpower or effortful inhibition (Holton 2009; Sripada 2020). These *process views* of self-control state that attempts at aligning behavior with commitments count as self-control only if they are produced by means of the right psychological processes. Others claim that any process that succeeds in aligning behavior with commitments counts as self-control. These *results views* imply that self-control can be realized by means of multiple different psychological processes (including effortful control, automatic processes, and habits), or even entirely realized in appropriately structured environments which generate the relevant results regardless of the involvement of intra-cranial psychological functions (Mele 1987, 2003; Heath and Anderson 2010; Vierkant 2014).^1^

As with many philosophical debates, most positions fall somewhere between these two extremes. Partisans of results views admit some causal constraints for behavioral alignment to count as self-control (Mele 2003), and some process views recognize that self-control sometimes consists less in the use of a cognitive capacity and more in its skillful deployment (Levy 2017). That said, the two theories make vastly different predictions about the conditions under which people can be rightly said to exercise self-control.

Suppose you have two friends who are trying to quit smoking. After smoking her last cigarette, one friend throws out all her cigarettes, tells everyone about it to enlist community support, and makes a pact with her co-workers that if they catch her smoking she will pay $2.000 dollars. The other friend resorts to using sheer willpower and distracts herself whenever she sees someone else smoking. She even keeps a pack of cigarettes in her work desk as a reminder that she’s the master of her own desires. Suppose your two friends have the same level of success. Who exercises self-control? If both do, does one exercise self-control to a greater extent?

According to the results view, both individuals exercise self-control equally. According to the process view, the first individual—the one who throws out her cigarettes—does not exercise self-control, since an individual exercises self-control only to the extent to which she resists the temptation with willpower, and willpower consists solely in intra-cranial psychological processes, not in external constraints on action. In fact, on the process view, it might seem that the first individual manipulates her environment to compensate for a perceived *lack* of self-control.

Noticeably absent from debates about the relative theoretical merits of process and results views is systematic evidence about the folk-psychological concept of self-control. This is somewhat surprising, as the two views make opposed predictions about the *extension* of the folk concept of self-control, as noted above. Additionally, while the results and process views may seem opposed, the folk concept of self-control might be structured in a way that reconciles the two. For example, people might have a prototype concept of self-control, with different instances of self-controlled behavior considered more prototypical (in some respect) relative to other instances. Moreover, research into the folk concept of self-control is of theoretical relevance also because folk-psychological constructs sometimes play a role in fixing the reference of theoretical terms in psychology and some areas of philosophy (Chihara and Fodor 1865; Nichols 2015; Vargas 2017), and some theorists hold that if a theory of self-control aligns with the folk view that speaks in favor of the theory (Sripada 2020; Levy 2017).

On the practical side, the extension and structure of the folk concept might influence how people frame opportunities to exercise self-control, as well as which tactics are available for them to select and implement. Evidence suggests people who conceive of self-control as relying on a depletable resource perform more poorly on self-control tasks relative to people who believe that self-control does not consume a limited resource (Job et al. 2010; Klinger et al. 2018). People’s conception of self-control could influence strategy selection by determining which strategies become accessible, thereby impacting performance. Given self-control’s significance for long-term wellbeing and health outcomes (Moffitt et al. 2011), it is of great interest to investigate whether the ‘folk’ concept of self-control aligns with scientific self-control research.

In this paper, we argue that folk psychological thinking about self-control partially aligns with both opposing views, but sides more closely with process views. Specifically, while the concept’s extension aligns with results views, the concept turns out to be prototypically structured, where the prototype aligns with process views. We report the results of two pre-registered behavioral studies that support these claims. The results suggest that while people recognize a plurality of strategies as genuine instances of self-control, purely internal exercises of self-control are more frequently proposed, more easily accessible, and considered more efficacious and more advisable than their externally-scaffolded counterparts. This implies a hierarchical structure for the folk psychological category of self-control. The concept encompasses a variety of regulatory strategies and organizes these strategies along a hierarchical continuum, with fully intra-psychic strategies at the center and fully scaffolded strategies at the periphery.

### Traditional Views of Self-Control

Despite the prevalence of philosophical theorizing about self-control and its importance in different Western religious traditions, there has been very little work done to understand how people attribute self-control to others. Typically, discussions of self-control attribution have investigated the conditions under which people think that a *failure* of self-control constitutes *weakness of will* (Doucet and Turri 2014; May and Holton 2012; Mele 2010; Newman et al. 2014; Rosas et al. 2018; Sousa and Mauro 2014). In what follows, we sketch several lines of evidence that indicate people are pluralists about what kinds of processes and strategies constitute self-control.

Some folk psychological commitments about self-control seem to emphasize results over processes. Externally-supported self-control strategies are those that rely on off-loading the need to resist temptation to the environment and to other people. These strategies have been shown to be more effective in increasing student academic success (Duckworth et al. 2016b); reducing high-calorie food consumption (Privitera and Zuraikat 2014); reducing alcohol consumption during residential treatment for alcohol use disorder (Soravia et al. 2015); increasing rates of smoking cessation among habitual smokers attempting to quit (Wagner et al. 2004); and helping people stay on an exercise program longer (Mazzoni et al. 2019)⁠. Many 12-Step self-help groups use principles of community support and situation management to address problems with substance abuse (Donovan et al. 2013). More generally, strategies involving a selection or alteration of the agent’s situation seem to be more effective and less costly than those relying on attention, working memory and inhibitory capacities (Duckworth et al. 2016a, b); goal-attainment success is correlated not with frequently resisting temptations but with feeling fewer temptations in the first place (Milyavskaya and Inzlicht 2017); and people with high trait self-control seem to avoid temptations rather than resist them (Hofmann et al. 2012). However, it should be acknowledged that purely intra-psychic strategies can sometimes be as effective at facilitating goal attainment as externally-supported strategies (Milyavskaya et al. 2020); that some specific situational strategies can be less effective than some specific intra-psychic strategies (Hennecke and Bürgler 2020); and that much research remains to be done to more clearly specify strategy effectiveness. That said, recent research indicates that intra-psychic strategies are susceptible to acute limitations and seldom used by those with higher trait self-control (Inzlicht and Friese 2021); and that the benefits of externally-supported strategies are far-reaching (Duckworth et al. 2018).

Resisting (and eventually overcoming) bad habits is commonly thought of as a key function of self-control, and the evidence cited above shows externally-supported strategies appear to facilitate this. Thus, to the extent that people are sensitive to the positive impact of these strategies for habit management, we think that they will be likely to view these strategies as instantiating self-control. This is a reason to consider the folk concept might align with results views: these strategies count as instances of self-control precisely *because* they lead to successful resistance and habit revision.

That said, while people might exhibit pluralism with respect to what counts as self-control, they might be biased toward thinking of purely internal exercises of self-control as more representative of the concept. A reason to suspect this for Western populations in particular is that intellectual and religious traditions tend to favor process views of self-control. The well-known charioteer metaphor from Plato’s *Phaedrus* (253c–254e) represents the view that self-control is an intrinsic feature of the individual’s soul—the soul’s rational element forcing its volitional element into alignment in the face of inner conflict. Moreover, Plato elsewhere reveals more straightforward commitments to a process view. In Book I of the *Laws*, the Athenian Stranger chastises his Cretan companion for Crete’s laws prohibiting the experience of great pleasures on the grounds that manipulating the environment to preclude the possibility of temptation also precludes developing the ability to *resist* temptation:

…if our citizens grow up without any experience of the keenest pleasures, and if they are not trained to stand firm when they encounter them, and to refuse to be pushed into any disgraceful action, their fondness for pleasure will bring them to the same bad end as those who capitulate to fear. Their slavery will be of a different kind, but it will be more humiliating: they will become the slaves of those who are able to stand firm against the onslaughts of pleasure who are past-masters in the art of temptation—utter scoundrels, sometimes. Spiritually, our citizens will be part slave, part free, and only in a limited sense will they deserve to be called courageous and free (*Laws* 635c-d).^2^

Plato thus acknowledges that we can sometimes deploy strategies to minimize exposure to temptation, thereby increasing our chances of behaving in accordance with our better judgment. But in this passage he claims these strategies do not *exemplify* a self-controlled character; rather, they *compensate* for a lack of it.

Process views are also found in the Christian tradition, where virtues require not only right action but also an appropriate orientation of mind. For example, the virtue of chastity requires temperance, where this implies that one “make moderate use of bodily members in accordance with the judgment of reason and the choice of will” (*Summa theologiae* IIaIIae Q. 151, a. 1). Similarly, Augustine notes that self-control is needed to overcome a recalcitrant will divided against itself (*Confessions* VII, 3.5).^3^

Thus, traditional perspectives emphasize the centrality and efficacy of effortful, intra-psychic self-control strategies. We therefore suspect that, either because traditional perspectives reify folk perspectives or because folk concepts reflect traditional theories, it is plausible that people incorporate the process flavor of these traditional theories. The influence of the process tradition might lead people to focus on intra-psychic strategies as prototypical cases, thus making externally-supported strategies less salient and less valued in everyday practical thinking.

Process views also permeate contemporary discussion, where philosophers and cognitive scientists argue that genuine self-control necessarily implies expending some non-trivial amount of mental effort (Holton 2009; Levy 2011; Shenhav 2017)⁠, and reject considering purely effortless, pre-emptive strategies as instances of self-control (Sripada 2020). Similar traces of the process view appear in the psychological tradition. William James, for one, considered that “effort of attention” is “the essential phenomenon of will” (James 1890, p. 562)⁠, and an influential psychological theory compared self-control to a mental muscle that depletes with continued use (Baumeister et al. 1998; Baumeister and Tierney 2012).^4^ The latter theory has received significant media attention, suggesting another way in which process views may be influencing the folk concept. Conversely, these theories may themselves be influenced by underlying assumptions about the nature of self-control as an internal trait. Either way, if there are communicating paths from theory to folk psychology, these would suggest that the folk concept of self-control would tend to consider intra-psychic exertions of self-control as prototypical instances of the kind.

### Overview

In the following pre-registered studies, we tested the following predictions: (1) People will recognize externally-supported strategies as instances of self-control. (2) People will recognize intra-psychic strategies as more representative of the concept of self-control. (3) People’s assessments of the value of externally-supported strategies will not coincide with the scientific evidence suggesting that externally-supported strategies are better; if anything, people will consider internal strategies as more effective than external strategies.

Evidence from Study 1 suggests that people recognize both intra-psychic and externally-scaffolded strategies as genuine exercises of self-control, although not to the same degree. Study 2 shows that people regard intra-psychic strategies as more effective, more advisable, and more salient than externally-scaffolded ones. Collectively, these results suggest that everyday thinking about self-control exhibits a mixture of process and results principles, although intra-psychic strategies are regarded as more prototypical.

## Study 1

### Methods and Materials

#### Participants

To determine sample size, we conducted an a priori power analysis using G*Power 3.1.9.6 (Faul et al., 2007). For a repeated measures ANOVA of five measures used to detect effect sizes that approximate those found in pilot studies (*f* = 0.709) with 99% power at a strict *p* value threshold (*p* < .001), the analysis suggested a sample size of 134 participants. To account for exclusions, we over-recruited by 10%. 151 participants voluntarily participated in this study on Prolific Academic (http://prolific.ac) for monetary compensation. 1 participant failed to pass the predetermined 2-min minimum time on the task, and 3 participants failed an attention check, so data were analyzed with the remaining 147 individuals (*M*_age_ = 31 years±10.3, range_age_ = [18,70], 106 females, 41 males). We analyzed data only after the required sample size target was met for all studies. De-identified data for all studies are available at https://osf.io/7ydph/?view_only=3640f4d6fa2b4486a23a3dab30d3c046.

#### Materials and Procedure

Each participant saw an initial *Temptation* situation:

*Temptation*: Taylor, Alex, Sam, Lee, and Jamie have a huge test in class tomorrow on a difficult topic. They all want to do well in class, so they need to study. But their friends are going out tonight, which should be a lot of fun.

Then participants saw five vignettes as a within-subjects independent variable that described the different self-control strategies each character deployed. Vignettes were presented in a random order.

- *Inhibition*: Taylor is tempted to go out, but she decides to study instead. Suddenly, her friends send a group text inviting people to come out to the bar. Taylor thinks it would be a lot of fun to go out now, but she knows deep down that she should study. So she makes the effort to reply that she won’t be able to go tonight. Although every now and then she feels tempted to go out, she resists and successfully keeps studying.

- *Reappraisal*: Sam is tempted to go out, but she decides to study instead. Suddenly, her friends send a group text inviting people to come out to the bar. She knows deep down that she should study. So instead of thinking about how much fun the bar will be, she remembers that the place is very loud, so whenever she goes there her ears buzz annoyingly for a couple of days. Then she looks at the material she’s studying and thinks ‘This is not so boring after all!’ Every time she feels tempted to go, she does this. And so she successfully keeps studying.

- *Attentional Distraction*: Lee is tempted to go out. She’s studying in a coffee shop on campus, and she’s around people who are talking about their fun evening plans. To distract herself from the conversations and focus on studying, she decides to put in her headphones and play her study playlist. When her friends send a text message inviting people to come out to the bar, Lee is so distracted by the music that she doesn’t notice the message. She keeps studying without interruptions.^5^

- *Situation Modification*: Alex is tempted to go out, but she activates a new app called StudyBuddy, which blocks your phone so that you cannot access any of it while you study. Her friends send a group text inviting people to come out to the bar, but the text doesn’t reach Alex because her phone is locked by StudyBuddy. Since she doesn’t know where her friends will be, she cannot go and meet them. Alex successfully keeps studying without interruptions.

- *Akrasia*: Jamie is tempted to go out, but she decides to study instead. Suddenly, her friends send a group text inviting people to come out to the bar. Jamie thinks it would be a lot of fun to go out now, but she knows deep down that she should study. However, she can’t stop thinking about how much fun it would be to go out tonight, so she gives up studying, gets ready and leaves for the bar.

Participants then answered five questions about the extent to which the character exerted effort, controlled her impulses, resisted temptation, displayed willpower, and displayed self-control. Participants registered their responses in a slider from 0 to 100 anchored at the midpoint (0 = not at all, 100 = entirely). These five questions correspond to different constructs commonly associated with self-control.

#### Hypotheses

We hypothesized that (1) there would be a significant difference in participant attributions of self-control between fully intrapsychic strategies (*Inhibition* and *Reappraisal* in this case) and externally supported strategies (*Self-Distraction* and *Situation Modification* in this case); and that (2) there would be no significant difference in self-control attribution between the two intrapsychic strategies.

## Results

**Fig. 1:**
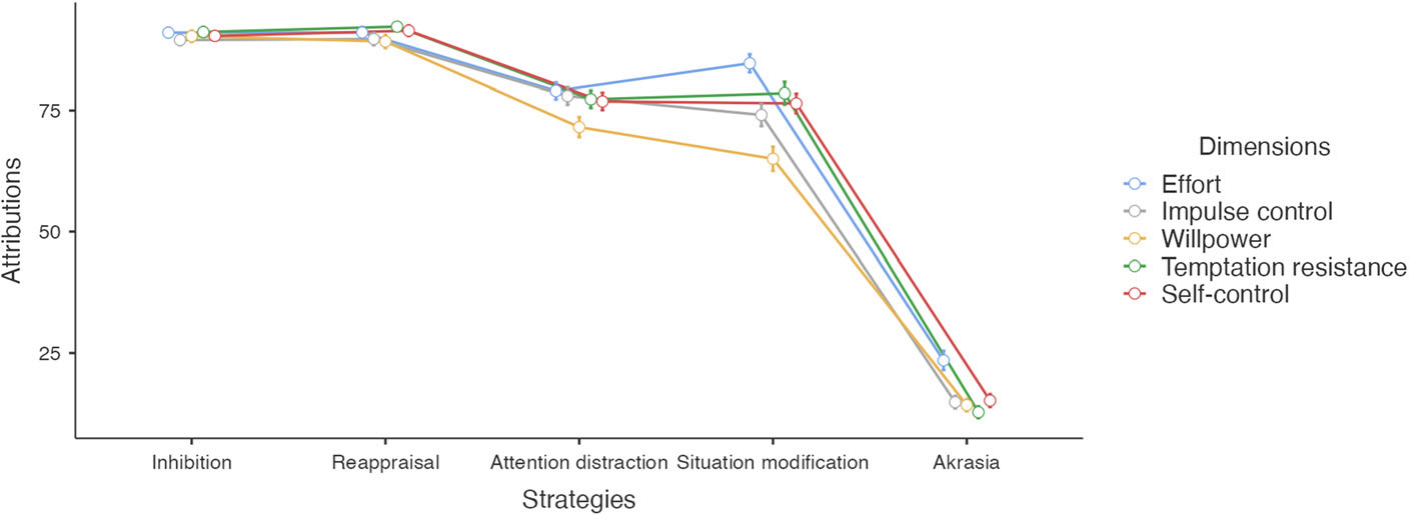
Attributions of self-control dimensions per strategy type. Error bars represent 95% confidence intervals

Figure 1 represents participant ratings of self-control dimensions across different strategies. (For means scores of self-control attributions see **Table E1** in the Supplementary Files.)

The five self-control dimensions (effort, willpower, impulse control, temptation resistance, and self-control) had strong internal reliability (α = 0.898, *95% CI* [0.88, 0.91]), which suggests strong inter-dependence among the different dimensions. An exploratory factor analysis fitting one dimension using maximum likelihood extraction showed that item responses for each dimension loaded onto a single factor (see Tables 1 and 2).^6^ A chi-square test indicated that one factor was sufficient to model the dimensions (χ^2^(5) = 28.3, *p* < .001). In light of these analyses, we computed a global self-control measure by taking the average score of item responses.

**Table 1:** Descriptive statistics for self-control ratings for each strategy

	Inhibition	Reappraisal	Attentional distraction	Situation modification	Akrasia
Mean	90.4	90.7	76.6	75.8	16.1
Standard deviation	10.8	11.7	19.0	21.0	14.3
Lower CL	87.9	88.1	74.0	73.2	13.5
Upper CL	93.0	93.3	79.1	78.3	18.7

*Note*. Confidence level used: 0.95

**Table 2.** Exploratory Factor Analysis of Self-Control Dimension responses

Dimension	Loading	Uniqueness
Effort	0.72	0.48
Impulse	0.77	0.41
Self-Control	0.87	0.25
Willpower	0.76	0.43
Temptation	0.85	0.28

**Note:** No rotation method was used because a single factor was extracted

A one-way ANOVA on average self-control attributions found a large effect of strategy (*F*(4, 730) = 556.2, *p* < .001, η^2^ = 0.75, 95% *CI* [0.67, 0.78]). However, this large effect size was likely due to the large difference between self-control attributions in the Akrasia condition and in the other conditions. To account for this, we ran a one-way ANOVA on average self-control attributions excluding responses from the Akrasia, which condition showed a smaller, but still large effect of strategy (*F*(3, 584) = 38.6, *p* < .001, η^2^ = 0.17, 95% *CI* [0.11, 0.22]).

Post-hoc comparisons between the strategies (Bonferroni-corrected for multiple comparisons) showed that strategies grouped into two categories: fully intra-psychic strategies (Inhibition and Reappraisal) were not significantly different. Similarly, externally-supported strategies (Self-Distraction and Situation Modification) were not significantly different. However, these two groups were significantly different from each other (*p* < .001) (Table 3). Such grouping suggests that intra-psychic strategies and externally-supported strategies are distinguished from each other, with the former eliciting greater attributions of self-control relative to the latter.

**Table 3:** Post Hoc Comparisons between self-control strategies

Comparison						
STRATEGIES		STRATEGIES	Mean Difference	df	t	*p*
Inhibition	–	Reappraisal	−0.268	290	−0.20	0.99
	–	Attention distraction	13.894	231	7.69	< .001
	–	Situation modification	14.683	218	7.52	< .001
Reappraisal	–	Attention distraction	14.162	242	7.68	< .001
	–	Situation modification	14.951	228	7.53	< .001
Attention distraction	–	Situation modification	0.789	289	0.34	0.98

*Note*. *p*-values are corrected for multiple comparisons using Tukey method

## Discussion

These results suggest that people group different strategies together according to their orientation (internal vs. external), and that they recognize both intra-psychic and externally-supported strategies as forms of self-control. Ratings of self-control for fully intra-psychic strategies (Inhibition and Reappraisal) strongly correlate, whereas ratings of self-control for externally-supported strategies (Self-Distraction and Situation Modification) strongly correlate. Fully intra-psychic strategies receive significantly higher ratings than externally-supported ones (Fig. 1), suggesting that intra-psychic self-control strategies are conceptualized as more prototypical exercises of self-control.

While Study 1 provides evidence that intra-psychic strategies manifest self-control to a greater degree, it has some limitations. It does not indicate a preference for intra-psychic strategies over externally-scaffolded ones. We want to know whether people consider intra-psychic strategies to be more prototypical also in an *evaluative* sense, i.e. whether they consider them to be more efficient or more choice-worthy than externally-scaffolded ones. And a higher rating of self-control does not necessarily indicate that. Moreover, our results could be the product of a demand effect. By asking participants to rate individuals in terms of effort, willpower, and control, we might have biased people toward internalist self-control strategies. Finally, while intrapsychic strategies have psychological costs, externally-supported strategies can have costs of other kinds (e.g. financial, reputational) that we did not measure in this study, and could be more clearly observed in a study that allowed participants to evaluate a broader set of strategies.

To overcome these limitations, we conducted another study where participants produced several self-control strategies for managing temptation using open responses. They also evaluated the effectiveness of the produced strategies and selected a single strategy that they would advise to someone managing the motivational conflict described in the vignette.

## Study 2

### Methods and Materials

#### Participants

To determine sample size, we ran an a priori power analysis using G*Power. For a chi-squared test to detect effect sizes approximating those found in pilot studies (*w* = .4) with 90% power at a strict *p* value threshold (*p* < .001), the analysis suggested 127 participants. To cover for possible exclusions, we aimed to recruit 140 participants. Because we had additional research funds, we recruited above this initial threshold. 164 participants voluntarily participated in this study on Prolific Academic for monetary compensation. 15 participants met our exclusion criterion (failing to provide self-control strategies three or more times), so data were analyzed with the remaining 149 individuals (*M*_age_ = 32 years±11.9, range_age_ = [18,65], 76 females, 73 males). No data was analyzed prior to stopping data collection.

#### Hypotheses

We hypothesized that (1) people would more frequently produce intra-psychic relative to externally-supported self-control strategies. Since prototypical instances are more immediately salient when employing a prototype concept (Margolis and Laurence 2019), we also predicted that (2) internal strategies would be produced earlier than external strategies. Further, we expected people to (3) rate intra-psychic strategies as more effective, and (4) advise them more frequently than external strategies.

#### Materials and Procedure

Each participant saw three vignettes describing a character facing a motivational conflict. Given the known effects of morality on self-control attributions (Rosas et al. 2018; Sousa and Mauro 2014)⁠, we varied the moral nature of the agent’s commitment. In the *neutral vignette*, someone’s boss is hosting a dinner party for a big client. The character needs to make a good impression, but the party is in a high-rise apartment building and the character is afraid of heights. In the *moral vignette*, a volunteer doctor is working in a remote location. She receives a patient and begins to feel nauseous at the sight of the patient’s gruesome injury, but she is the only qualified doctor on staff and needs to finish the operation to insure the patient’s survival. In the *immoral vignette*, the character is trying to enter a criminal gang. At a meeting with the boss, the character is overcome with fear in the presence of the leader’s dogs. However, she wants to make a good impression to enter the gang. (Pre-registration and complete materials are available in the supplementary files: https://osf.io/7ydph/?view_only=3640f4d6fa2b4486a23a3dab30d3c046.)

After each vignette, participants were asked: “What can [Name] do? Describe 3 different ways in which [Name] could try to exert self-control and stick to her commitment.” Following these open responses, participants were asked two additional questions: (1) “For each one of the options you mentioned, how effective is it as a self-control strategy?” and (2) “What would you advise the character to do?” Participants answered the first question using a slider from 0 to 100 anchored at the midpoint (0 = extremely ineffective, 100 = extremely effective). Participants answered Question 2 by selecting a single strategy from among those they had produced. After selecting which strategy to advise, participants were asked: “Why would you suggest that option rather than the others?” Finally, to explore the association between willpower and effort on strategy selection and evaluation, we asked participants to tell us, for each of the strategies produced, how much effort or willpower is required to implement the strategy, to which they responded using a slider from 0 to 100 anchored at the midpoint (0 = no effort/willpower, 100 = maximum effort / all the available willpower).

#### Coding Open Responses

Participant responses were classified along two dimensions:

1. *Orientation*: If implementing the self-control strategy requires only internal psychological resources, the strategy was classified as *internal (=intra-psychic)*. If implementing the strategy requires using features of the agent’s environment, it was classified as *external (=externally-scaffolded)*. To be maximally conservative with respect to our hypotheses, we classified strategies as fully intra-psychic only when they did not involve any external support for their implementation. If participants advised characters to give up, the strategy was labelled as *Akrasia*. If participants advised a strategy that violated the narrative constraints of the vignette, the strategy was labelled with *X*.
2. *Strategy type*: Strategy type classifications are based on Duckworth et al. (2016a, 2016b) taxonomy. Each strategy could be classified as *inhibition*, *cognitive reappraisal*, *attentional*, *or situational*. Attentional strategies were further subdivided in *attentional focus* and *attentional distraction*.

It is important to keep these two dimensions distinct: strategy orientation is about the *location* of the resources used to deploy a strategy (internal vs. external), while strategy type is about the *kind of process* involved in strategy deployment (e.g. directing one’s attention, changing one’s cognitive representations, or modifying one’s environment). While situational strategies are always externally-scaffolded, all other strategy types can be internally or externally oriented (e.g. I may distract myself by imagining a future vacation, or by playing music through my headphones).

Independent raters blind to the study’s hypotheses used classification instructions (available in the Supplemental Material) to sort participant responses. Inter-rater reliability was strong for both Orientation (91.6% agreement) and Strategy Type (80.9% agreement). Raters met to autonomously solve discrepancies and produce a unified categorization, which was then used for the analyses reported below.

To ensure our annotations captured the existing structure of the textual data participants provided, we compared them to classifications generated by topic modelling algorithms (see Online Supplemental Materials, section D). This revealed a convergence between rater classifications and automatically inferred classifications, suggesting that rater coding effectively tracks variances in the data and has a very low degree of arbitrariness.

## Results

1. ***Intra-psychic strategies are more frequently generated than externally-supported strategies.***

**Fig. 2:**
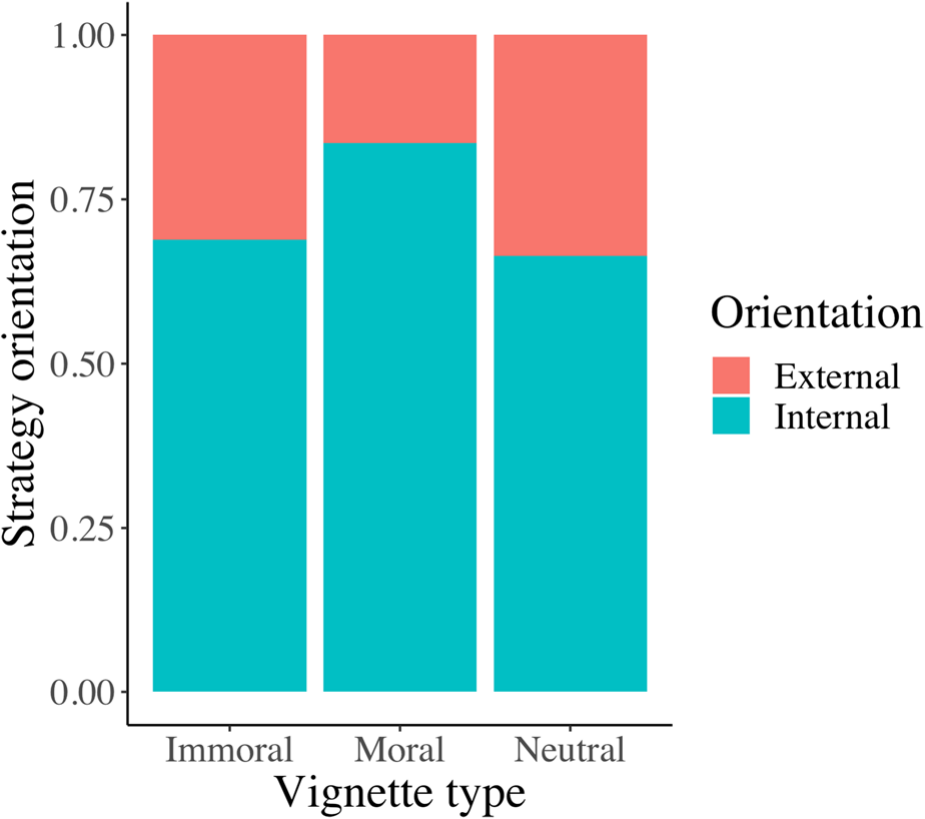
Proportions of Internal/External orientation in each vignette

Across all vignette types, people produced almost three times more internal than externally-supported strategies. A chi-square test indicated a moderate association between vignette type and strategy (χ^2^(1) = 261.78, *p* < .001, Cramer’s *V* = 0.46, 95% *CI*[0.40, 0.51]) (See Table 4). Internal strategies were more prevalent across all vignette types, though significantly more so in the moral vignette (χ^2^(2) = 36.24, *p* < .001, Cramer’s *V* = 0.17, 95% *CI*[0.11, 0.22]) (see Fig. 2).

**Table 4.** Strategy type (intra-psychic vs. externally-supported) per vignette type (immoral, moral, neutral)

	Immoral	Moral	Neutral	Total
Intra-psychic	294	341	284	919
Externally-supported	133	67	144	344

(2). ***Intra-psychic strategies are more salient***

**Fig. 3:**
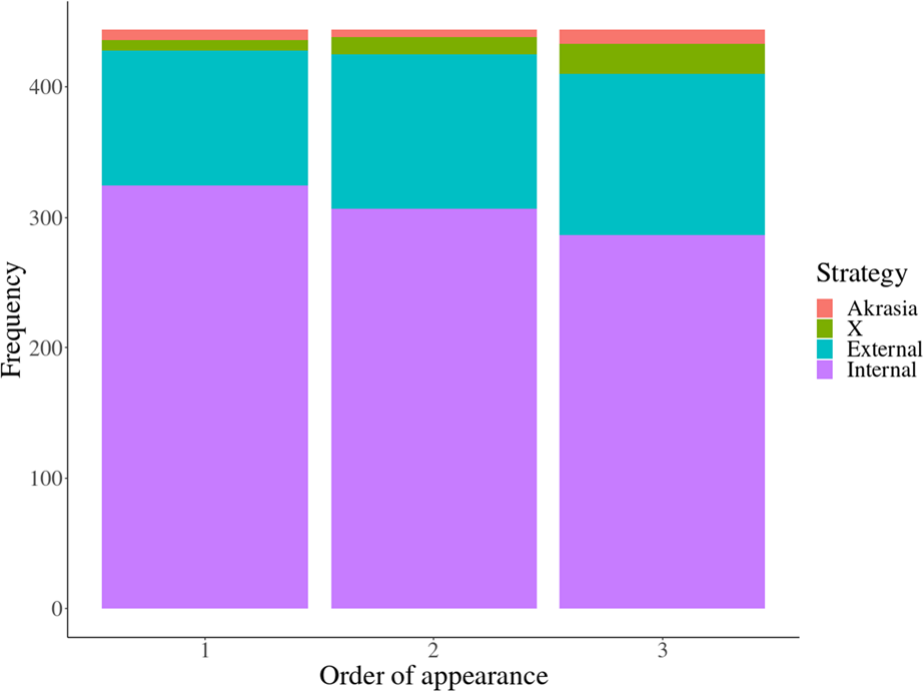
Frequency of internal strategies decreases by order of appearance, whereas external strategies and invalid answers increase. Invalid answers are those that advise the character to give up (labelled ‘Akrasia’), or responses that do not make sense in the context of the vignette (labelled ‘X’)

While participants produced significantly more internal strategies, we found that participants tended to produce more external strategies later in each block, with internal strategies decreasing and external strategies increasing from the first to the third intra-block attempt at strategy generation. When excluding invalid responses (Akrasia and X), the difference between block attempts only approached significance (χ^2^(2) = 3.81, *p* = .15). However, invalid answers also increase later in the block (**see** Fig. 3), and when these are included in the analysis, there is a significant difference in strategy frequency between attempts (χ^2^(6) = 13.72, *p* = .033). That said, the effect is small (Cramer’s *V* = 0.07, 95% *CI*[0.0, 0.10]) and the confidence interval includes 0.

(3). ***Intra-psychic strategies are advised more frequently***

People advise internal strategies more than twice as often as external strategies (I = 291, E = 141), and a chi-square test showed a moderate association between advised strategy and strategy orientation (χ^2^(1) = 52.08, *p* < 0.001, Cramer’s *V* = 0.35, 95% *CI* [0.25, 0.44]). Additionally, the moral valence of the situation significantly alters the kinds of strategies people advise. Indeed, in the moral situation, people advised more internal strategies than in the immoral situation. Also, the number of external strategies increased in the immoral and neutral situations (Table 5). However, even in these situations, people still advise a preponderance of internal strategies relative to external strategies, though the associations range from strong to weak (in the moral situation: χ^2^(1) = 45.88, *p* < .001, Cramer’s V = 0.57, 95% *CI*[0.40, 0.73]; in the immoral situation: χ^2^(1) = 4.05, *p* = .044, Cramer’s *V* = 0.17, 95% *CI*[0.00, 0.33]; in the neutral situation: χ^2^(1) = 13.77, *p* < .001, Cramer’s *V* = 0.31, 95% *CI*[0.14, 0.47]).

**Table 5:** Type of advised strategy (intra-psychic vs. externally-supported) per vignette type

	Immoral	Moral	Neutral	Total
Externally-supported	59	31	51	141
Intra-psychic	83	112	96	291

(4). ***Intra-psychic strategies are rated as significantly more effective***

We conducted a *t*-test to compare mean rating of effectiveness by strategy orientation. We computed a Welch’s *t*-test because responses were not normally distributed (W = 0.94, *p* < .001) and variance across groups was unequal (*f*(918, 343) = 0.81, *p* = 0.02). Further, because our hypothesis predicted a specific direction of difference in means between the two groups, we ran a one-tailed *t*-test to assess whether mean ratings of the effectiveness of intra-psychic self-control strategies was greater than externally-supported strategies. Results supported this hypothesis, though the effect was small and the confidence interval contains 0 (*t*(563.47) = −1.78, *p* = 0.037, *d* = 0.12, 95% *CI*[−0.006, 0.24]).

## Results from Exploratory Analyses

### Effort and Willpower Ratings

Results of a two-tailed Welch’s *t*-test showed that participants considered intra-psychic strategies to require more effort (*M* = 74.8, *SD* = 22.9, *n* = 919) than externally-supported strategies (*M* = 59.8, *SD* = 27.7, *n* = 344), with a large effect of strategy orientation on judgments of effort (*t*(528.93) = 8.96, *p* < .001, *d* = 0.62, 95% *CI*[0.49, 0.74]). Results of a two-tailed Welch’s *t*-test showed that participants also considered intra-psychic strategies to require more willpower (*M* = 77.1, *SD* = 21.5, *n* = 919) than externally-supported strategies (*M* = 61.6, *SD* = 27.3, *n* = 344), with a large effect of strategy orientation on judgments of willpower (*t*(510.63) = 9.52, *p* < .001, *d* = 0.67, 95% *CI*[0.54, 0.80]).

To assess the relationship between judgments of willpower and effort and ratings of effectiveness, we fitted linear models of effectiveness on willpower and effort. The model accounted for 8% of the variance in effectiveness ratings (*F*(2, 1329) = 57.46, *p* < .001, *R*^2^ = 0.08). Both judgments of willpower (β = 0.15, *p* < .001) and effort (β = 0.14, *p* < .001) had significant partial effects in the model. This indicates that for every unit increase in judgments of willpower and effort, ratings of effectiveness *increased* about 6.7 units. While this is a small effect, it is consistent with the mean differences observed between ratings of effectiveness for intra-psychic and externally-supported strategies. Intra-psychic strategies are judged to require more effort and willpower to implement, and yet, despite their higher costs, they are also considered more effective.

### Attentional Strategies Are Prevalent among Intra-Psychic Strategies

We classified strategies according to the taxonomy in Duckworth et al. (2016a, 2016b)⁠: situation modification, attentional choice, cognitive reappraisal, and inhibition. We divided attentional choice into two further categories: self-distraction and attentional focus. *Self-distraction* consists in turning attention away from features pertaining to the tempting stimuli and the self-controlled action, whereas *attentional focus* consists in maintaining attention on goal-relevant features. Attentional distraction was the most common strategy selection, while cognitive reappraisal was the least common (Table 5). When attentional strategies (Focus and Distraction) are combined, the attentional category accounts for nearly half of all strategies (47%, *n* = 634) (Table 6).

**Table 6:** Strategy type (intra-psychic vs. externally-supported) per vignette type (immoral, moral, neutral)

	Immoral	Moral	Neutral	Total
Attention-distraction	128	116	149	393
Inhibition	127	101	71	299
Situation modification	94	55	102	251
Attention-focus	45	114	82	241
Reappraisal	33	22	24	79

**Table 7:** Summary of coefficients for multinomial regression model of Strategy Type on Vignette Morality

Strategy type	Immoral	Moral	Neutral
*Situation Modification*	0.72(*0.16*)	0.52(*0.20*)	1.69(*0.19*)
*Cognitive Reappraisal*	0.24(*0.24*)	0.18(*0.30*)	0.31(*0.31*)
*Attention Focus*	0.37(*0.20*)	1.12(*0.17*)	1.33(*0.20*)
*Attention Distraction*	1.01(*0.15*)	1.19(*0.16*)	2.16(*0.18*)

*Note.* Values are exponentiated odds ratios (log likelihood). Parenthetical values are standard errors of odds ratios. All coefficients are significant predictors in the model (*p* < .001) as calculated using a 2-tailed Wald chi-squared test on standardized log likelihood values. Reference level for strategy type = Inhibition

### Effects of Morality

We found a small but significant association between the vignette’s moral valence and strategy orientation. Intra-psychic strategies were significantly more prevalent in the Moral than in the Immoral and Neutral vignettes (Fig. 2). Morality also played a role in the strategy type produced by participants⁠ (Table 6). Compared to the other two vignettes, the Immoral vignette contained fewer attentional strategies: attentional strategies accounted for 56% of the total in the Moral vignette and 54% in the Neutral vignette, but they were only 40% of the total in the Immoral vignette. Comparing only Moral and Immoral vignettes, while self-distraction was the most prevalent strategy in both, attentional focus was much more prevalent in the Moral than in the Immoral vignette.

To better understand the relationship between strategy type and morality, we ran a multinomial logistic regression to predict the likelihood of strategy selection based on vignette using the *nnet* package in R (Venables and Ripley 2002). Coefficients in the model are odds ratios that estimate changes in the likelihood of the outcome variable (e.g., Strategy Category) based on predictors in the model (e.g., Vignette Morality). Our model included only one nominal predictor variable (Vignette Morality). Unlike ordinary least squares regression, there is no measure of explained variance (*R*^2^) for logistic regression. However, there are several approximations of *R*^2^ associated with logistic regression. We computed the Nagelkerke modified pseudo-*R*^2^, which can be interpreted as a measure of the predictive strength of the model relative to a model that contains no predictors (i.e., an ‘intercepts-only’ or empty model). The model containing Vignette Morality as a predictor had a pseudo-*R*^2^ value of 0.062, and the predictive value of the model was significantly greater than the empty model (LR χ^2^(8) = 53.3, *p* < .001).

For interpretation, we exponentiated the coefficients of the model to represent the log likelihood of changes in Strategy based on Vignette Type. The reference level of the outcome was set to Inhibition, so each coefficient represents the likelihood of selecting a strategy based on vignette type relative to the probability of selecting inhibition in that same vignette type (see Table 7 for summary of model coefficients).

The model indicates that participants are less likely to advise cognitive reappraisal strategies over inhibition across all vignette types (likely driven by the small number of cognitive reappraisal strategies produced). Participants were more likely to select Inhibition strategies over Situation Modification strategies in either the Immoral or Moral vignette, but more likely to select attentional strategies over inhibition in the Moral vignette. Moreover, all of these coefficients had significant partial effects in the model (all *p* < .001). Based on these likelihood estimates, the model predicts a Strategy type for each observation based on Vignette type. To test model accuracy, we compared model predictions with actual observations. The model achieved 38.9% accuracy. While this might seem low, note that this is well above chance performance (5 strategy types = 20% performance at chance) and is based on a single predictive factor (Vignette Morality). This suggests that the moral valence of the situation depicted in the vignette plays a significant role in the kind of self-control strategy selected.

## Discussion

Study 2’s results strongly suggest that people tend to generate more intra-psychic strategies to manage motivational conflicts relative to externally-supported strategies. The wide prevalence of internal strategies, and the small but significant increase in externally-supported strategies in later responses, suggests that internal strategies are significantly more salient in practical thinking about self-control: self-control is strongly associated with intra-psychic processes, making externally-supported strategies less available when devising solutions for a self-control conflict.

People also tend to advise intra-psychic strategies more frequently than externally-supported strategies, and tend to consider the former more effective. This is evidence that intra-psychic strategies tend to be evaluatively preferred to externally-supported strategies. The higher advisability and effectiveness of intra-psychic strategies might be a function of greater accessibility and centrality of such strategies to the concept of self-control. However, we did not find a large effect, and further experiments are needed to replicate and possibly qualify this result.

We also found that judgments of the willpower and effort needed to implement a strategy predict ratings of strategy effectiveness. Stronger judgments of willpower and effort are positively associated with ratings of effectiveness. This aligns with the finding that intra-psychic strategies are advised more frequently than externally-supported strategies.

In the discussion of Study 1, we suggested that asking participants about the effort and willpower needed to implement a strategy might bias participants. Specifically, we thought that by cueing participants to think about the amount of effort and willpower associated with a strategy, they would tend to generate more intra-psychic strategies since they may be thought to require more willpower and effort. In order to mitigate this risk, effort and willpower questions appeared last in the questionnaire for each vignette, i.e. participants saw them only after they had provided their strategies and evaluated their advisability and effectiveness. The presence of these questions did not have the expected biasing effect, since participants tended to produce more intra-psychic strategies during the first attempt in each vignette (before they encountered the questions) and fewer intra-psychic strategies in subsequent attempts (after having encountered the questions at least once).

Although we did not have an explicit hypothesis about this, one may reasonably expect that strategies considered to require lower amounts of willpower and effort would be considered more effective and advised more frequently. But we found the opposite: participants advised strategies that are perceived to be more effortful and require more willpower to a greater extent, and these strategies were also considered more effective.

Collectively, and corroborating the findings from Study 1, these results suggest that people have a robust tendency to conceptualize self-control in a prototypical fashion, where intra-psychic, and particularly attentional, processes are central traits of self-control. This prototypical structure explains the greater frequency, salience, and evaluative superiority of intra-psychic strategies. Study 2’s results thus jointly suggest that, while the extension of the folk concept of self-control is compatible with results views (i.e. externally-supported strategies are included as genuine methods for exerting self-control), the folk concept nevertheless exhibits a structure predominantly aligned with process views, since purely intrapsychic cases are treated as prototypical instances of the concept both descriptively (as suggested by their availability) and evaluatively (as suggested by effectiveness and advisability ratings), even despite being considered more psychologically costly (by requiring greater exertions of effort and willpower).

## General Discussion

Together, these studies identify central features of everyday thinking about self-control. We made two broad predictions: (1) attributions of self-control would exhibit a mixture of results and process views about self-control; and (2) the structure of the folk concept would coincide more closely with process views: intra-psychic exercises of self-control would be considered more prototypical than externally-supported strategies. Our studies support both predictions, suggesting that the folk concept is prototypically a process view despite also including elements aligned with results views.

According to these findings, folk thinking about self-control diverges from both process and results views, reflecting a hybrid of the two views. The boundaries of the concept coincide with results views, as externally-scaffolded regulatory strategies are considered genuine instances of self-control. But the structure of the concept coincides with process views, according a central place to strategies that recruit only intra-psychic processes.

This cuts against the view suggested by some researchers that in everyday thinking self-control is nothing more than effortful resistance (or what Levy (2017) calls “direct control”; see also Holton’s (2009, p. 127) discussion of effort in relation to strength of will). That said, this does not *settle* any conceptual debates about the most perspicuous characterization of self-control. Folk psychological categories do not necessarily carve nature at its proverbial joints, and folk thinking might exhibit systematic error in the kinds of phenomena believed to be instances of self-control (Sripada 2017). That said, we do think that folk psychological categories can often provide parameters for how to fix the referents of terms in our theories (Vargas 2017).

In that regard, the folk concept of self-control’s novel structure should be considered in future theorizing. Distinctions reflected in current accounts of self-control might, and seemingly do, fail to appropriately capture the ontology of self-regulation (Eisenberg et al. 2019; Herdova 2017; Inzlicht et al. 2020), so the situation is ripe for conceptual innovation. The results here suggest an alternative to traditional conceptual structures, one that encompasses a variety of regulatory strategies and organizes them along a hierarchical continuum, with fully intra-psychic strategies as central and fully scaffolded strategies at the periphery. This provides novel inspiration for future reflection, considering that theories consistent with this structure would have the advantage of agreement with the common-sense view. The view’s merits and drawbacks should be tested and discussed in future work on the topic.

Our results indicate multiple directions for future work. First, if it is true that people tend to prefer intrapsychic strategies, this might manifest in people selecting such strategies to manage real-world motivational conflicts. An important question that follows from this is whether intrapsychic strategies *should* in fact be preferred. While the effectiveness of a strategy depends significantly on various contextual factors and cannot be judged in the abstract (Bonanno and Burton 2013), we have earlier mentioned some preliminary evidence suggesting that externally-scaffolded strategies tend to be more effective (while also mentioning the limitations of this evidence). In particular, situational strategies that intervene at earlier stages of the regulatory process (i.e., pre-empting rather than resisting the feeling of temptation) are the most likely to successfully issue in goal persistence.

Add to this that the tendency to produce attentional strategies reveals a limited repertoire of regulatory strategies, over-reliance on which has been associated with greater risk of psychopathology (Bonanno and Burton 2013; Lougheed and Hollenstein 2012). Thus, there are practical reasons for using behavioral and psychophysiological tools to further assess the scope of this preference for intrapsychic strategies, as well as whether people display such preference also when facing real-life self-control conflicts.

The interactions between strategy selection and moral valence also merit further investigation. Exploratory analyses from Study 2 suggest that the motivational conflict’s moral valence is associated with strategy selection. In a moral context (i.e. a situation where the agent had morally praiseworthy intentions and sought to perform a morally praiseworthy action), people produced considerably more attentional focus strategies than in an immoral context. One possible explanation for this is that in advising certain strategies, people believe that being aware of the goodness of some activity (when it is considered morally good) is sufficient for motivating the agent to do the activity. This reflects a kind of motivational internalism in cases where people have morally praiseworthy intentions (Björklund et al. 2012). The implication is that selecting an externally-scaffolded strategy for performing good actions could reveal a moral flaw because it suggests one is unable to be sufficiently motivated just by appreciating the moral goodness of one’s commitment. For good people, goodness should be enough of a motivating factor. When commitments are immoral, however, focusing on them provides no additional motivational force, so attentional distraction becomes a more viable alternative. If true, this would explain why morally praiseworthy commitments are associated with a prevalence of attentional focus strategies for managing motivational conflicts, while attentional distraction strategies are equally prevalent for morally bad commitments. Further, if this reflects everyday thinking about self-control, then the preferential bias toward intrapsychic strategies would be expected when people engage in moral self-improvement. However, confirmatory evidence is needed to support this proposal, since evidence so far is only exploratory.

Future studies should move beyond the limitations of the present research. As the relationship between morality and strategy selection shows, situational factors can alter how people think about self-control. Thus, beliefs about different strategies might be context-sensitive, raising issues about generalizability. To assess the robustness of judgments about self-control dimensions and self-control attributions, the relationships between strategies and situations should be systematically investigated. In Study 2, all the vignettes presented the agent already immersed in a situation and close to the moment of action. This could have biased the strategies suggested by participants. In future work vignettes should manipulate the framing of the vignette, to capture different stages of the regulatory process in which the agent finds herself, and examine whether this has an effect on strategy production and evaluation. Additionally, some studies have found that people tend to select early-disengagement strategies like distraction to regulate high-intensity stimuli, and tend towards late-engagement strategies like reappraisal to regulate low-intensity stimuli (Murphy and Young 2018; Sheppes et al. 2014). Since all Study 2 vignettes involved high-intensity stimuli, this could explain the prevalence of distraction and the low levels of reappraisal as suggested strategies. Thus, future studies should include more varied vignettes to further corroborate that the patterns reported here are generalizable.

Notably, these limitations might indicate a context-sensitive element in everyday thinking about self-control. If the framing of vignettes influences deliberation about self-control strategies, then perhaps the kinds of strategies deployed are a function of how the situation is framed. Several factors might explain this. One is that some self-control strategies rely on the use of imaginative faculties such as episodic simulation and counterfactual thinking (Schacter et al. 2012; Watkins 2008). In some cases, reliance on imagination is straightforward: cognitive reappraisal, for example, consists in imaginatively reframing one’s perception of a tempting stimuli. In other cases, imagination contributes more indirectly. Situational modification strategies, for instance, rely on understanding how to reorganize one’s environment to pre-empt the experience of temptation altogether, which requires imagining how to recombine environmental elements and simulating the effectiveness of this recombination. Some situation framings might make certain possibilities more remote, thereby making it more difficult to imaginatively engage these scenarios. Different ways of framing the same situation (or varying the temporal scale of presenting the situation) might induce different strategies or make certain possibilities more salient. The preference for intrapsychic strategies might, then, be reduced or even overcome with a little nudging that frames motivational conflicts in ways that incline people to consider more externally-scaffolded strategies.

## Supplementary Information

ESM 1 (PDF 45.5 kb)

## Footnotes

1 The process views / results views labelling was proposed by Sripada (2020).

2 Plato (1970), tr. Trevor J. Saunders.

3 An interesting exception to the Western process tradition is Nietzsche, who outlines six different modes of “combating the vehemence of a drive” including: “avoid[ing] opportunities for gratification of the drive” and “deliberately giv[ing] oneself over to the wild and unrestrained gratification of a drive in order to generate disgust with it” (*Daybreak* 109). Needless to say, Nietzsche recognizes a rich variety of strategies as legitimate forms of self-control that maps in interesting ways onto contemporary results views of self-control.

4 This trend, however, seems to be turning recently, favoring the possibility of effortless, environmentally-outsourced self-control. For recent reviews, see Amaya (2020) and Inzlicht et al. (2020).

5 This strategy could be seen as blurring into situation modification (putting on headphones arguably counts as modifying one’s environment). We developed Study 2 to be able to distinguish more clearly between each strategy type. The crucial point here is that both *Attentional Distraction* and *Situation Modification* portray instances of externally-supported strategies, as opposed to *Inhibition* and *Reappraisal*, which are purely intra-psychic.

6 The optimal number of factors to extract was determined using the nScree function in the *nFactors* package in R (Raiche 2010). The function returns the results of Cattel’s Scree test and the acceleration factor of eigenvalues for the items. Both are compared to the number of factors extracted by parallel analysis. In this case, all tests indicated that one factor should be extracted (see Supplementary Files).
