# Supplementary material for: What’s inside is all that counts? The contours of everyday thinking about self-control
Source: Rev Philos Psychol. 2021 Sep 30;14(1):33–55. doi: 10.1007/s13164-021-00573-2 (PMC10033625; doi:10.1007/s13164-021-00573-2)
Supplement: Supplementary file 1 — (PDF 45.5 kb) [file 13164_2021_573_MOESM1_ESM.pdf]

Dear Springer Support Team,

I write about two things related to the proofs of my paper "What's inside is all that counts? The contours of everyday thinking about self-control". I send this attached (and also by email) because for some reason the comments function within the eProofing system isn't allowing me to break spaces between lines.

Thank you and all best,
Juan Pablo Bermúdez

1. HEADINGS

Regarding AQ3 in the proofs to this article, I needed to point out that section heading levels have multiple issues. They should correspond precisely to this structure:

1. Introduction
1.1. Traditional views of self-control
1.2. Overview

2. Study 1
2.1. Methods and materials
2.1.1. Participants
2.1.2. Materials and procedure
2.1.3. Hypotheses
2.2. Results
2.3. Discussion

3. Study 2
3.1. Methods and materials
3.1.1. Participants
3.1.2. Hypotheses
3.1.3. Materials and procedure
3.1.4. Coding open responses
3.2. Results
3.2.1. Intra-psychic strategies are more frequently generated than externally-supported strategies
3.2.2. Intra-psychic strategies are more salient
3.2.3. Intra-psychic strategies are advised more frequently
3.2.4. Intra-psychic strategies are rated as significantly more effective
3.3. Results from exploratory analyses
3.3.1. Effort and willpower ratings
3.3.2. Attentional strategies are prevalent among intra-psychic strategies
3.3.3. Effects of morality
3.4. Discussion

4. General discussion

The title and headings would have only the first letter of the sentence capitalized, if possible.

Since this issue is so delicate, I would ask you to please send me a new version of the proofs after these corrections have been made. (I promise I will reply quickly!)

2. ACKNOWLEDGEMENTS

Could you add to the paper an acknowledgments section with this paragraph?

The authors would like to thank Santiago Amaya, Zachary Irving, Kevin Reuter, and Chandra Sripada for detailed feedback and comments at different stages of the writing process. We also thank audiences at the University of Michigan, the Empirical Philosophy Lab at the University of Pittsburgh, and the XPhi Europe 2020 conference. This publication was made possible through the support of the John Templeton Foundation grant #61255, and the Swiss National Science Foundation's project The Nature and Value of Efforts.
